# Unilateral diaphragmatic paralysis following cervical spine surgery in a patient with neurological symptoms: a case report and review of the literature

**DOI:** 10.3389/fphys.2026.1685728

**Published:** 2026-02-16

**Authors:** Kai Chen, Jiafu Zhu

**Affiliations:** Department of Orthopedic Spine Surgery, Tongde Hospital of Zhejiang Province, Hangzhou, Zhejiang, China

**Keywords:** cervical myelopathy, cervical spondylosis, diaphragmatic paralysis, dyspnea, phrenic nerve

## Abstract

**Rationale:**

Unilateral diaphragmatic paralysis is an extremely rare postoperative complication following cervical spine surgery. We present a case of unilateral diaphragmatic paralysis following cervical spine surgery in a patient with preoperative neurological symptoms and discuss the related literature.

**Patient concerns:**

A 74-year-old male presented with gait instability and frequent falls. Postoperatively, the patient developed dyspnea.

**Diagnoses:**

Postoperative imaging revealed right-sided diaphragmatic elevation, indicating phrenic nerve dysfunction. Cervical myelopathy at C3/4 and C4/5 levels was identified as a relevant underlying condition during the determination of the postoperative course.

**Interventions:**

The patient underwent anterior cervical discectomy and fusion (ACDF) at C3/4 and C4/5. Conservative management, including oxygen therapy and neurotrophic support, was provided postoperatively.

**Outcomes:**

Significant improvement in respiratory symptoms was observed by postoperative Day 42, with a reduction in right hemidiaphragm elevation on chest computed tomography (CT). The patient’s dyspnea gradually improved with treatment, and respiratory function stabilized.

**Lessons:**

This case highlights the need to monitor for respiratory complications after cervical spine surgery, even in patients without preoperative respiratory symptoms. Neurological signs should prompt early recognition of phrenic nerve involvement to prevent further complications and improve outcomes.

## 1 Introduction

Unilateral diaphragmatic paralysis is a rare condition associated with various etiologies, including cervical spondylotic pathology, particularly when the phrenic nerve or its originating nerve roots are involved. Clinically, it may present with respiratory symptoms and, in some cases, be accompanied by neurological manifestations such as radicular pain or motor weakness (Ricoy et al., 2019; O’Beirne et al., 2019; Lamb et al., 2024). However, unilateral diaphragmatic paralysis has been reported in the absence of overt neurological symptoms, particularly in postoperative or postprocedural settings (Ricoy et al., 2019). The phrenic nerve, primarily originating from the C3, C4, and C5 nerve roots, plays a crucial role in diaphragm function, with the C4 nerve root contributing most to its motor control (Fuller et al., 2022). In cervical spine surgeries, especially those involving the C3/4 level, there is a potential risk of damage to the C4 nerve root, leading to phrenic nerve dysfunction (Gonzalez et al., 2023).

Cervical spondylosis is among the most common pathological processes leading to degenerative cervical myelopathy (DCM), which is recommended by the AO Spine Knowledge Forum as the overarching term for degenerative conditions of the cervical spine resulting in spinal cord dysfunction (Ganau et al., 2018). DCM encompasses a broad spectrum of clinical manifestations that may extend beyond classical motor and sensory deficits to include less recognized complications involving adjacent neural structures.

Previous case reports have documented the association between cervical spondylosis and diaphragmatic paralysis, but most cases included typical neurological signs (Kitamura et al., 2022; Park et al., 2020). This case report describes a rare instance of unilateral diaphragmatic paralysis in a patient with cervical spondylotic myelopathy following anterior cervical discectomy and fusion (ACDF) surgery. Despite having no preoperative respiratory symptoms or significant neurological deficits, the patient developed postoperative dyspnea, which was later attributed to phrenic nerve dysfunction, possibly related to intraoperative or postoperative involvement of the C4 nerve root. The patient experienced persistent chest tightness and shortness of breath, which were initially unexplained. However, these symptoms were eventually linked to progressive right hemidiaphragm elevation observed on chest computed tomography (CT), confirming diaphragmatic paralysis. The C4 nerve root, which primarily controls the diaphragm, was likely affected during surgery at the C3/4 segment, possibly contributing to the postoperative respiratory complications (Yang et al., 2024).

This case underscores the clinical importance of considering unilateral diaphragmatic paralysis as a rare postoperative complication following cervical spine surgery in patients with cervical spondylotic pathology, even in the absence of overt neurological symptoms. It also highlights the significance of timely diagnosis and appropriate surgical intervention. Furthermore, this case emphasizes the necessity of using more accurate diagnostic tools beyond chest CT for the early recognition and diagnosis of phrenic nerve injury.

## 2 Case report

A 74-year-old male presented to the neurology department with complaints of gait instability in both lower limbs for 1 year. Upon admission, he was diagnosed with Parkinson’s disease and sleep disorders. The patient reported approximately six falls per day. After cervical spine magnetic resonance imaging (MRI) was performed, a consultation with the spinal surgery department was requested. Based on the patient’s preoperative neurological status, including gait instability, upper limb weakness, and positive pathological reflexes, the modified Japanese Orthopedic Association (mJOA) score was retrospectively calculated as 13 points (Martin et al., 2021), indicating moderate degenerative cervical myelopathy.

**FIGURE 1:**
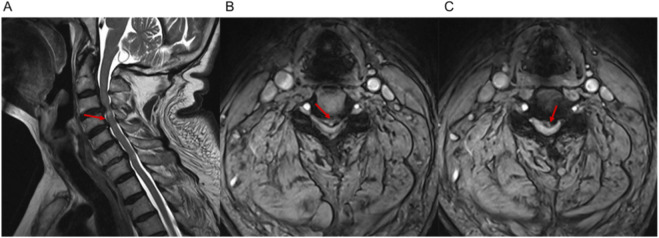
Preoperative cervical spine MRI showing disc protrusions and stenosis at C3/4 and C4/5 levels. **(A)** Sagittal view showing signal changes in the spinal cord at the C4 level, with significant disc protrusions and stenosis at C3/4 and C4/5 levels. **(B)** Axial view of the C3/4 intervertebral disc showing disc protrusion and canal narrowing. **(C)** Axial view of the C4/5 intervertebral disc showing similar findings of disc protrusion and stenosis.

Upon physical examination, Hoffman’s sign was positive bilaterally, and the patient’s grip strength was grade 4 in both upper limbs. Cervical spine MRI revealed signal changes in the spinal cord at the C4 level, with disc protrusions and significant stenosis at the C3/4 and C4/5 levels (Figures 1A–C). Based on these findings, a diagnosis of cervical myelopathy was made, and surgical intervention was recommended. The patient was transferred to the spinal surgery department for further evaluation.

**FIGURE 2:**
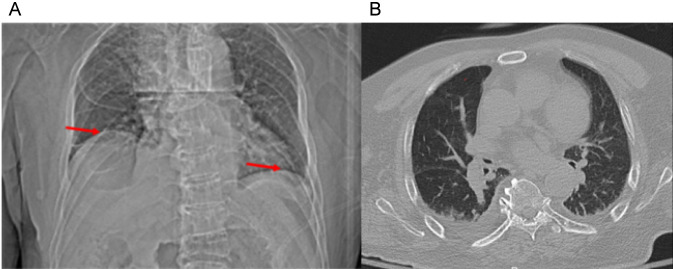
Chest computed tomography on postoperative Day 3. **(A)** Scout view demonstrating early elevation of the right hemidiaphragm. **(B)** Axial CT image (lung window) showing minimal bilateral pleural effusion and scattered pulmonary infiltrates.

After completing preoperative assessments, the patient underwent ACDF at the C3/4 and C4/5 levels. On the third postoperative day, the patient developed chest tightness and dyspnea, though vital signs, blood gas analysis, and oxygen saturation were normal. CT revealed minimal pleural effusion bilaterally, with scattered areas of infection (Figure 2). Despite intravenous administration of dexamethasone, the patient’s respiratory symptoms did not improve significantly. He was subsequently treated with cephalosporins for infection control, theophylline for bronchospasm relief, and methylprednisolone for inflammation. His chest tightness and dyspnea partially improved but continued to recur intermittently, accompanied by a productive cough with white, foamy sputum.

**FIGURE 3:**
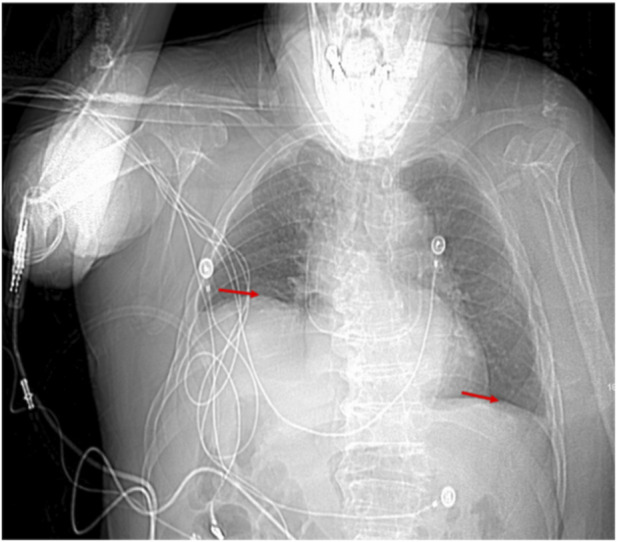
Chest CT (scout view) on postoperative day 5 showing further elevation of the right hemidiaphragm.

By postoperative Day 5, the patient was transferred to the respiratory department for further management. That night, he experienced worsening chest tightness and difficulty breathing, leading to a diagnosis of respiratory failure, and he was transferred to the intensive care unit for high-flow oxygen therapy. A repeat chest CT showed significant elevation of the right hemidiaphragm, likely due to phrenic nerve edema or paralysis (Figure 3). Neurotrophic therapy was initiated, and after stabilization, the patient was transferred back to the respiratory department on postoperative Day 7.

On postoperative Day 10, the patient continued to experience episodic chest tightness and dyspnea, mainly at night, lasting 5–6 h per episode. Sleep difficulties and psychological distress emerged, with the patient expressing concerns about death. A psychiatric consultation was arranged, and medication was prescribed to aid sleep. Over time, the patient began developing mental health issues.

On postoperative Day 16, the patient reported weakness in the left limbs, with a muscle strength of grade 2 in the left upper limb, grade 3 in the left lower limb, grade 4 in the right upper limb, and grade 5- in the right lower limb. Electromyography (EMG) was performed, which indicated neurogenic damage in both the upper and lower limbs, suggesting nerve root involvement. Bilateral pathological reflexes were positive. A neurology consultation suggested a possible sympathetic response secondary to spinal cord involvement (C3–C5). On postoperative Day 20, a multidisciplinary team (MDT) discussion took place, and although delayed hemorrhage and soft tissue edema in the neck were considered, subsequent cervical spine MRI and other examinations were ordered to exclude these possibilities.

EMG results on postoperative Day 25 revealed neurogenic damage in both the upper and lower limbs. A second MDT discussion on postoperative Day 28 reviewed the patient’s chest CT images taken on postoperative Days 3, 5, and 22, respectively. Notably, the right hemidiaphragm elevation corresponded with worsening respiratory symptoms and partial recovery as symptoms improved. The patient’s respiratory difficulties were concluded to be related to diaphragm dysfunction and likely associated with phrenic nerve involvement. Rehabilitation therapy was recommended.

**FIGURE 4:**
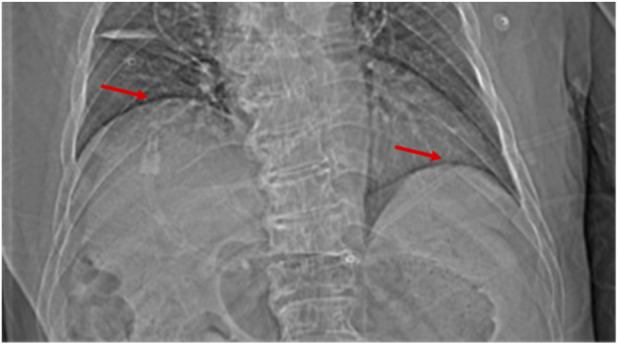
Chest CT (scout view) on postoperative day 22 showing further elevation of the right hemidiaphragm.

**FIGURE 5:**
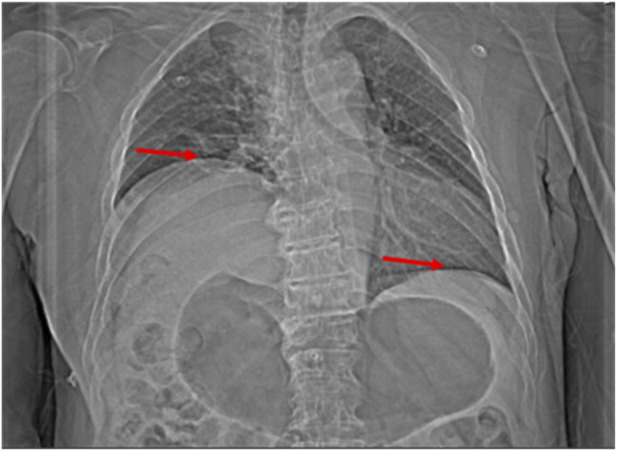
Chest CT (scout view) on postoperative day 42 showing a continued elevation of the right hemidiaphragm compared to earlier scans.

By postoperative Day 42, although the patient’s dyspnea had gradually improved, chest CT showed further elevation of the right hemidiaphragm, suggesting continued phrenic nerve dysfunction (Figures 4, 5). The patient was discharged on postoperative Day 52 with stable respiratory function.

## 3 Discussion

Unilateral diaphragmatic paralysis is an uncommon but clinically significant condition associated with cervical spondylotic pathology and, more rarely, as a postoperative complication following cervical spine surgery (Lamb et al., 2024; Kitamura et al., 2022). In this case, the patient, who initially presented with neurological symptoms such as gait instability in both lower limbs and frequent falls, developed right-sided diaphragmatic paralysis postoperatively. Although neurological symptoms have often reported in conjunction with respiratory dysfunction in previous studies, our patient had no respiratory symptoms before surgery. This highlights the importance of careful postoperative respiratory monitoring, even in patients who present primarily with neurological manifestations.

The postoperative development of dyspnea in this patient was likely multifactorial. Early postoperative chest CT demonstrated minimal bilateral pleural effusion and scattered pulmonary consolidations, which may have contributed to transient respiratory symptoms. In addition, prolonged bed rest and reduced mobility during the intensive care unit stay may have further exacerbated dyspnea. However, these factors alone cannot fully explain the persistence, nocturnal predominance, and fluctuating nature of the patient’s respiratory distress. Notably, serial chest CT scans revealed progressive elevation of the right hemidiaphragm that closely correlated with the onset, worsening, and partial improvement of respiratory symptoms, supporting diaphragmatic dysfunction as a major contributing factor. Phrenic nerve dysfunction was therefore considered the most plausible mechanism underlying the unilateral diaphragmatic paralysis, potentially related to intraoperative or postoperative involvement of the C4 nerve root. This is consistent with the findings of Park et al. (2020), who described a case of unilateral diaphragmatic paralysis secondary to cervical foraminal stenosis involving the C4 nerve root, in which the patient presented with exertional dyspnea in the absence of overt neurological deficits. In our case, chest CT demonstrated elevation of the right hemidiaphragm, supporting the diagnosis of diaphragmatic paralysis, and the patient’s gradual recovery following neurotrophic therapy and respiratory management further underscores the significance of early recognition and timely intervention.

Early recognition and management of phrenic nerve dysfunction are crucial for preventing prolonged respiratory distress and complications. Anekar et al. (2021) reported that delayed diagnosis of diaphragmatic paralysis due to cervical pathology can lead to prolonged respiratory distress and complications. In contrast, our case demonstrates that timely diagnosis, supported by MDT discussions and imaging, can prevent further deterioration and lead to favorable outcomes. The patient’s diaphragmatic elevation progressively resolved over time, corroborating the findings of Kaufman et al. (2021), who reported that surgical decompression and appropriate respiratory management can result in significant recovery of diaphragmatic function.

One of the key implications of our study is the need for postoperative monitoring of respiratory function in patients undergoing cervical spine surgery. While chest CT is helpful for detecting diaphragmatic elevation, more precise tools, such as pulmonary function tests or electromyography, should be considered for comprehensive diaphragmatic function assessment. Other studies, such as those of El Helow et al. (2024), have demonstrated the utility of these tools in diagnosing phrenic nerve involvement. The incorporation of these diagnostic tools in future cases may lead to earlier detection of phrenic nerve damage, allowing the timely implementation of more targeted interventions.

Future research should focus on exploring the mechanisms underlying phrenic nerve involvement in patients with cervical spondylosis. Specifically, investigating the role of nerve root compression and its potential impact on respiratory function could help refine surgical approaches and postoperative care protocols. Additionally, the potential for phrenic nerve recovery with conservative treatments such as neurotrophic therapy, as observed in this case, warrants further investigation to optimize treatment strategies for patients with similar presentations (Kaiser et al., 2017).

This case broadens the clinical spectrum of DCM by demonstrating that unilateral diaphragmatic paralysis may occur as a rare postoperative complication following cervical spine surgery, even in patients without preoperative respiratory symptoms. Rather than representing a direct manifestation of cervical spondylosis itself, this complication is more likely related to perioperative involvement of the phrenic nerve or its originating nerve roots within the context of DCM. A multidisciplinary approach involving spinal surgeons, respiratory specialists, and rehabilitation teams is crucial for ensuring comprehensive care and timely intervention. Future research should aim to standardize the management of diaphragmatic paralysis and explore more advanced diagnostic and monitoring methods beyond chest CT. Importantly, this case suggests that unilateral diaphragmatic paralysis should be considered a rare postoperative complication following cervical spine surgery rather than a direct manifestation of cervical spondylosis itself.

## Data Availability

The original contributions presented in the study are included in the article/supplementary material, further inquiries can be directed to the corresponding author.

## References

[B1] Anekar A. A. Nanjundachar S. Desai D. Lakhani J. Kabbur P. M. (2021). Case report: late-presenting congenital diaphragmatic hernia with tension gastrothorax. Front. Pediatr. 9, 618596. 10.3389/fped.2021.618596 33937144 PMC8081028

[B2] El Helow M. R. Ahmed S. F. El Bably M. M. ElHadba D. M. (2024). Neuromuscular phrenic nerve ultrasound versus nerve conduction study of phrenic nerve in amyotrophic lateral sclerosis. QJM An Int. J. Med. 117 (Suppl. ment_1), hcae070.537. 10.1093/qjmed/hcae070.537

[B3] Fuller D. D. Rana S. Smuder A. J. Dale E. A. (2022). The phrenic neuromuscular system. Handb. Clinical Neurology 188, 393–408. 10.1016/B978-0-323-91534-2.00012-6 35965035 PMC11135908

[B4] Ganau M. Holly L. T. Mizuno J. Fehlings M. G. (2018). Future directions and new technologies for the management of degenerative cervical myelopathy. Neurosurg. Clin. N. Am. 29 (1), 185–193. 10.1016/j.nec.2017.09.006 29173432

[B5] Gonzalez G. A. Miao J. Porto G. Harrop J. (2023). Bilateral phrenic nerve palsy after posterior cervical decompression and fusion surgery: a rare event after surgery. Spinal Cord Ser. Cases 9 (1), 41. 10.1038/s41394-023-00595-1 37573432 PMC10423263

[B6] Kaiser R. Ullas G. Havránek P. Homolková H. Miletín J. Tichá P. (2017). Current concepts in peripheral nerve injury repair. Acta Chir. Plast. 59 (2), 85–91. 29446308

[B7] Kaufman M. R. Chang E. I. Bauer T. Rossi K. Elkwood A. I. Paulin E. (2021). Phrenic nerve reconstruction for effective surgical treatment of diaphragmatic paralysis. Ann. Plastic Surg. 87 (3), 310–315. 10.1097/SAP.0000000000002896 34397519

[B8] Kitamura K. Hayashi H. Ishibashi R. Toda H. (2022). Recovery from hemidiaphragmatic paralysis with improved respiratory function following cervical laminoplasty and foraminotomy: illustrative case. J. Neurosurg. Case Lessons 4 (15). 10.3171/CASE22282 36461835 PMC9552678

[B9] Lamb C. D. Schupper A. J. Quinones A. Zhang J. Y. Steinberger J. Margetis K. (2024). Cervical spine stenosis causing diaphragmatic paralysis: a case study and narrative review of clinical presentations and outcomes. Clin. Spine Surg. 37 (6), 245–251. 10.1097/BSD.0000000000001588 38419161

[B10] Martin A. R. Jentzsch T. Wilson J. R. F. Moghaddamjou A. Jiang F. Rienmueller A. (2021). Inter-rater reliability of the modified Japanese orthopedic association score in degenerative cervical myelopathy: a cross-sectional study. Spine (Phila Pa 1976) 46 (16), 1063–1069. 10.1097/BRS.0000000000003956 33492085

[B11] O’Beirne S. L. Chazen J. L. Cornman-Homonoff J. Carey B. T. Gelbman B. D. (2019). Association between diaphragmatic paralysis and ipsilateral cervical spondylosis on MRI. Lung 197, 727–733. 10.1007/s00408-019-00271-y 31535202

[B12] Park H.-Y. Kim K.-W. Ryu J.-H. Lim C.-R. Han S.-B. Lee J.-S. (2020). Cervical foraminal stenosis causing unilateral diaphragmatic paralysis without neurologic manifestation: a case report and review of the literature. Medicine 99 (37), e21349. 10.1097/MD.0000000000021349 32925710 PMC7489730

[B13] Ricoy J. Rodríguez-Núñez N. Álvarez-Dobaño J. Toubes M. Riveiro V. Valdés L. (2019). Diaphragmatic dysfunction. Pulmonology 25 (4), 223–235. 10.1016/j.pulmoe.2018.10.008 30509855

[B14] Yang M. Zhong N. Dai Z. Ma X. Leng A. Zhou Y. (2024). Risks for prolonged mechanical ventilation and reintubation after cervical malignant tumor surgery: a nested case–control study. Eur. Spine J. 33, 1–13. 10.1007/s00586-024-08313-7 38907855

